# The dual role of Piezo1 in tumor cells and immune cells: a new target for cancer therapy

**DOI:** 10.3389/fimmu.2025.1635388

**Published:** 2025-07-31

**Authors:** Peng Qu, Hongyan Zhang

**Affiliations:** ^1^ Department of Anesthesiology, Chengdu Wenjiang District People’s Hospital, Chengdu, China; ^2^ Institute of Cardiovascular Diseases & Department of Cardiology, Sichuan Provincial People’s Hospital, School of Medicine, University of Electronic Science and Technology of China, Chengdu, China

**Keywords:** Piezo1, mechanosensitive ion channel, cancer immunotherapy, tumor microenvironment, immune modulation

## Abstract

Piezo1, a mechanosensitive ion channel, plays a pivotal and multifaceted role in tumor progression, immune evasion, and therapeutic resistance by transducing extracellular mechanical stimuli—such as matrix stiffness and fluid shear stress—into intracellular calcium influx. In tumor cells, Piezo1 promotes proliferation, invasion, and metastasis by activating oncogenic signaling and contributes to an immunosuppressive TME through regulation of cancer-associated fibroblasts (CAFs) and extracellular matrix (ECM) remodeling. In the immune compartment, Piezo1 integrates mechanical cues with metabolic and epigenetic reprogramming to orchestrate the functions of T cells, macrophages, and natural killer (NK) cells. Notably, Piezo1 deficiency impairs TH9 cell differentiation, diminishes T cell cytotoxicity, and enhances the activity of regulatory T cells (Tregs). Furthermore, Piezo1 expression correlates with distinct tumor immune phenotypes, such as “cold tumors,” and with responses to immunotherapy, making it a promising predictive biomarker for treatment efficacy. Given its dual regulatory roles in tumor biology and immune modulation, targeting Piezo1—such as through combination with programmed death-1 (PD-1) blockade—offers a potential strategy to reverse immunosuppression and enhance antitumor immunity. This review summarizes emerging insights into Piezo1’s role in cancer progression and immune regulation and highlights its translational potential as a novel target in cancer immunotherapy.

## 1 Introduction

Cancer incidence and mortality rates continue to rise globally, making malignancies the second leading cause of death after cardiovascular diseases and posing a significant threat to public health (1). Tumorigenesis is a multifactorial and multistage pathological process, among which immune evasion plays a pivotal role in cancer progression. Under normal physiological conditions, the immune system can recognize and eliminate abnormal cells. However, tumor cells develop diverse strategies to escape immune surveillance, thereby facilitating their growth and metastasis (2). This immune escape not only contributes to the primary development of tumors but also profoundly influences metastasis, therapeutic resistance, and the efficacy of immunotherapies (3). By remodeling the immune microenvironment, tumor cells induce immune tolerance and avoid immune-mediated destruction. This process is closely linked to immune system dysfunction and presents considerable challenges to the success of cancer immunotherapy (4–6).

Notably, the immune system within the tumor microenvironment (TME) exhibits a dual role: on one hand, it holds the potential to eliminate malignant cells; on the other, it can be co-opted by tumor cells to promote their survival, proliferation, and dissemination. This intricate interplay between tumor cells and immune components underscores the complexity of tumor immunoregulation and has become a focal point in current cancer research. According to the distribution and abundance of cytotoxic immune cells within the TME, tumors can be classified into three major immune phenotypes: immune-inflamed, immune-excluded, and immune-desert. Immune-inflamed tumors, also referred to as “hot” tumors, are characterized by high levels of T cell infiltration, upregulated expression of programmed death-ligand 1 (PD-L1), and elevated tumor mutational burden (TMB), making them more responsive to immune checkpoint inhibitors. In contrast, tumors that transition toward immune-excluded or immune-desert states are referred to as “altered” and “cold” tumors, respectively (7). Against this backdrop, increasing attention has been directed toward Piezo1, a mechanosensitive ion channel, for its emerging roles in both tumor biology and immune regulation. As a key mechanotransducer, Piezo1 responds to extracellular physical stimuli—such as membrane stretch and fluid shear stress—by modulating intracellular calcium (Ca²^+^) signaling, thereby influencing various cellular behaviors (8–11). In the context of tumor biology, Piezo1 contributes to malignant phenotypes including tumor proliferation, metastasis, and therapy resistance by modulating the mechanical properties and adaptability of cancer cells (12–14). For instance, Piezo1-mediated mechanotransduction helps tumor cells sustain survival advantages under hostile microenvironmental conditions, thereby accelerating tumor progression (15). In terms of immune regulation, Piezo1 is broadly expressed across a variety of immune cells, including macrophages, T cells, NK cells, and dendritic cells (DCs), and functions as a fine-tuned regulator of immune cell activity through mechanosensory pathways. Specifically, in T cells, Piezo1 is directly involved in facilitating TCR–pMHC interactions and promoting the formation of immune synapses, thereby enhancing T cell activation (16, 17). In B cells, Piezo1 is essential for recognizing membrane-bound antigens, and its deficiency leads to impaired B cell activation (18). In NK cells, Piezo1 activation enhances cytotoxicity and tumor infiltration capacity (19). Furthermore, in macrophages, Piezo1 regulates tumor-associated macrophages (TAMs) polarization and functional reprogramming by modulating inflammatory responses and mechanotransduction (20–23). Collectively, these findings highlight the multifaceted role of Piezo1, not only as a driver of tumor progression but also as a key modulator of immune cell function, thereby contributing to tumor immune evasion.

In summary, Piezo1, as a mechanosensitive ion channel, has emerged as a cutting-edge focus in cancer research due to its pivotal roles in tumor initiation, progression, and immune regulation. Its direct functions in tumor cells, along with its modulatory effects on immune cell behavior, offer novel insights into the mechanisms of tumor immune evasion. Moreover, these findings lay a theoretical foundation for the development of Piezo1-targeted cancer therapies, potentially opening new avenues for immunotherapeutic intervention.

## 2 Structure and function of the Piezo1 ion channel

Mechanosensitive ion channels are a class of key molecular sensors that efficiently convert mechanical forces into electrochemical signals (24). The Piezo protein family consists of Piezo1 and Piezo2, with the functional unit composed of a homotrimer made up of three identical subunits. Each subunit contains 38 transmembrane helices, which are arranged asymmetrically to form a unique propeller-like structure. In the plane of the membrane, the Piezo channel adopts a distinctive nano-bowl conformation, a structural feature that underpins its mechanosensory function (25). Among them, Piezo1, also known as Fam38A, is the core component of mechanically activated ion channels (8). Since its discovery by the team of Ardem Patapoutian, Piezo1 has become a focal point of research due to its critical role in mechanosensation. Patapoutian’s groundbreaking work on Piezo1 was recognized with the Nobel Prize in Physiology or Medicine in 2021.

### 2.1 Structure and function of Piezo1

**Figure 1:**
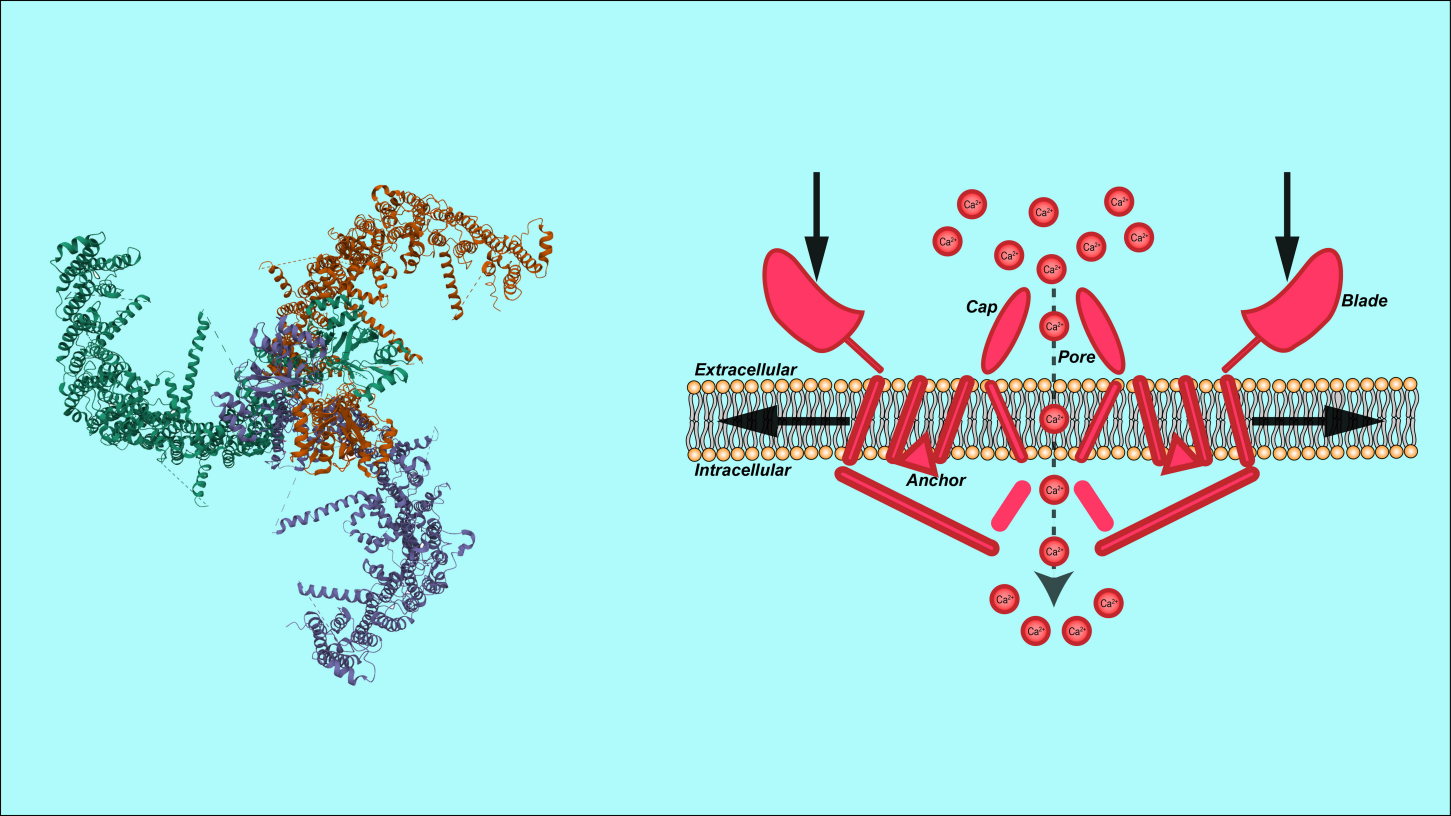
Structural representation of Piezo1 channel. Showing its 3D structure (left) and membrane interaction (right), including key components: cap, pore, blade, and anchor, with calcium ion (Ca²^+^) involvement.

Piezo1 is a highly conserved transmembrane protein consisting of 2,547 amino acid residues and exhibiting a wide range of functional characteristics. Its unique structure enables it to respond to mechanical stimuli and transduce signals. The basic functional unit of Piezo1 is a homotrimer of approximately 900 kDa, which adopts a distinctive propeller-like shape composed of three blades. This structure includes several key components, such as the curved blades, beam-like structures, and cap-like structures. The central channel of is composed of extracellular helices, an extracellular C-terminal domain, intracellular helices, and an intracellular C-terminal domain (25). The peripheral region of Piezo1 consists of 2,200 amino acids, encompassing the extracellular blade region, peripheral transmembrane helices, intracellular beam structure, and anchoring region (25, 26). The blade-like structure is formed by multiple transmembrane segments, providing sufficient flexibility to undergo deformation under external mechanical forces, which in turn affects the gating state of the ion channel (24, 27). Each blade is connected to the central channel through the transmembrane region, enabling the transfer of mechanical force to open the channel pore, allowing ions to pass through (28). The trimeric structure of Piezo1 functions through its peripheral mechanotransduction domains and the central ion channel pore. The beam-like structures, which are positioned at an approximately 30° angle to the membrane plane, act as levers. They couple the distal blades to the central pore, converting external mechanical signals into intracellular ionic conduction signals (29). Specifically, the activation of Piezo1 involves partial flattening of the blades, bending of the beams, and detachment and rotation of the cap. These changes collectively lead to the opening of the ion conduction path, allowing the passage of non-selective cations such as Ca²^+^, Na^+^, and K^+^ (30). Notably, Ca²^+^, as an important second messenger, plays a crucial role in cellular signal transduction, regulating processes such as cell growth, migration, differentiation, and apoptosis (31)(As shown in **Figure 1**).

Yoda1 is a widely used synthetic small molecule Piezo1 agonist. It binds to the mechanosensitive arm region of Piezo1, inducing conformational changes that open the channel pore, thereby accelerating the transport of Ca²^+^ through Piezo1 (32). This mechanosensitive arm region is located between two transmembrane domains of Piezo1, near the channel pore (33). By modulating the gating conformation of the channel, Yoda1 lowers the mechanical activation threshold, allowing the channel to be partially activated even in the absence of external mechanical stimuli (34). In contrast, Grammostola mechanotoxin 4 (GsMTx4) is a peptide secreted by the tarantula Grammostola that specifically inhibits the activity of Piezo1 and other mechanosensitive ion channels (35). Its primary mechanism of action is not direct binding to the gating elements of the Piezo1 channel, but rather through altering local membrane tension, which prevents Piezo1 from responding to external mechanical signals (36).

**Figure 2:**
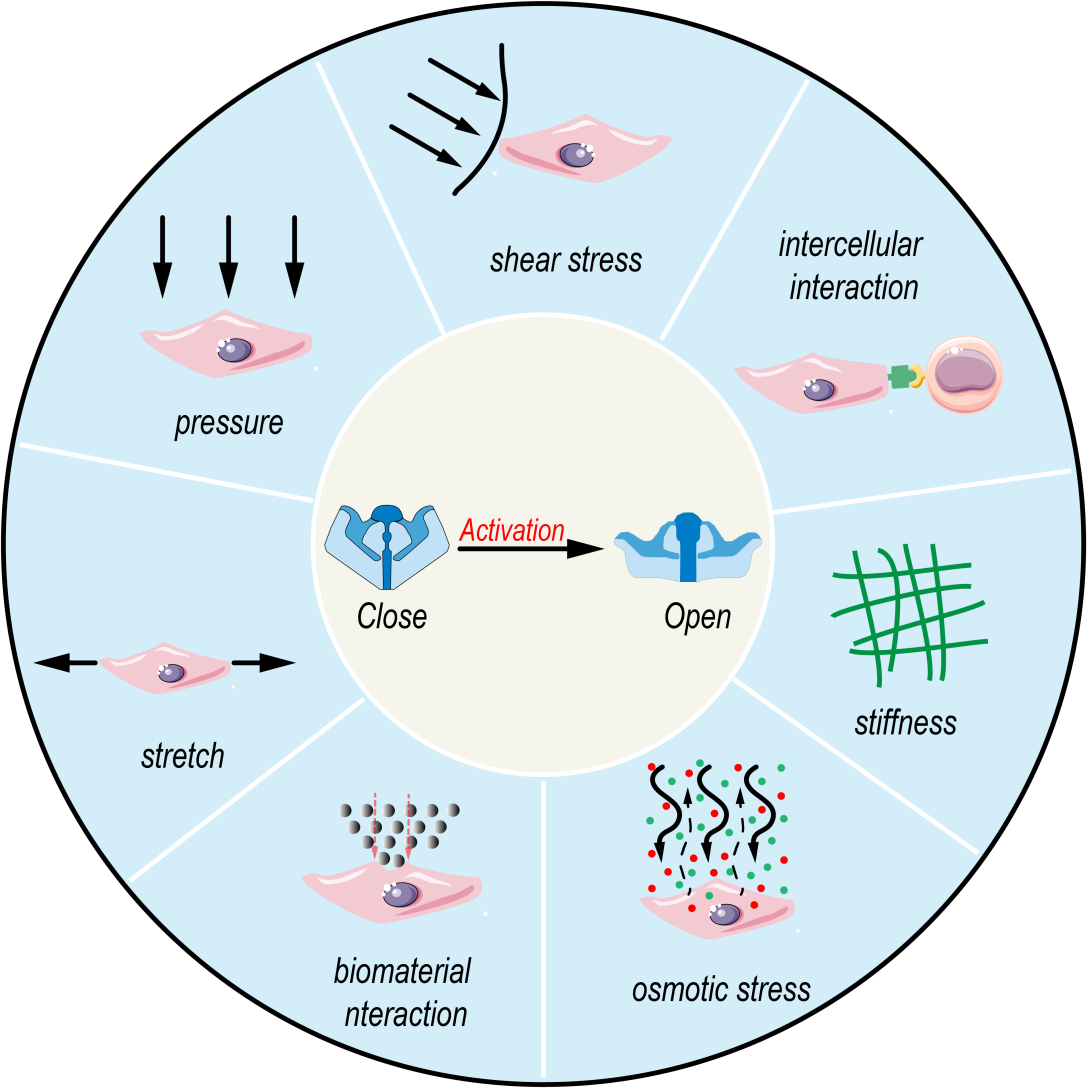
Activation of Piezo1 by multimodal biophysical stimuli. Various mechanical and chemical cues, including pressure, shear stress, intercellular interaction, stiffness, osmotic stress, biomaterial interaction, and stretch, activate mechanosensitive ion channels like Piezo1, leading to channel opening from a closed state.

Overall, Piezo1, as a mechanosensitive ion channel, responds to forces at the piconewton (pN) level (37), Its unique homotrimeric structure enables it to sense extracellular mechanical stimuli, such as membrane stretch, shear flow, changes in matrix stiffness, and tissue compression, as well as endogenous chemical signals (38), These stimuli activate Piezo1, leading to the influx of Ca²^+^, which in turn participates in the regulation of multiple physiological processes (39) (As shown in **Figure 2**). When mechanical force is applied to the cell membrane, Piezo1 undergoes conformational changes that trigger Ca²^+^ ion influx, while also inducing dynamic reorganization of the F-actin network. The resulting tensile stress further amplifies channel activity, establishing a positive feedback loop for mechanosignal regulation (38). Moreover, the amplitude and duration of Ca²^+^ signaling serve as a “fingerprint” that determines the specificity of Piezo1-mediated signaling: transient and sharp Ca²^+^ peaks are more likely to engage nuclear factor of activated T cells (NFAT)-dependent immediate early genes, whereas sustained and gradual elevations tend to couple with amplifier networks such as Yes-associated protein–TEA domain transcription factor (YAP–TEAD) or AKT–mechanistic target of rapamycin (AKT–mTOR). Relevant scenarios will be elaborated in the subsequent sections.

### 2.2 Piezo1’s functional diversity

Piezo1 plays a crucial role in sensing external mechanical forces, but its intracellular functions also exhibit a broad diversity. The activation and function of Piezo1 show significant heterogeneity across different cell types, particularly evident in its distinct mechanisms of action in tumor cells, immune cells, and stromal cells.

Piezo1 in Tumor Cells: Dysregulated function of Piezo1 in tumor cells has been closely associated with tumor initiation, progression, and metastasis. As tumor cells undergo changes in their mechanical properties, these alterations may lead to aberrant activation of Piezo1, which in turn promotes tumor cell proliferation, metastasis, and drug resistance (12–14). By sensing mechanical cues in the TME—such as increased tissue stiffness and compression— Piezo1 influences the proliferation and migration of tumor cells, thereby contributing to tumor progression (40, 41). Moreover, abnormal expression of Piezo1 may enhance the immune evasion capacity of tumor cells by modulating immune cell function, further impacting tumor immune escape mechanisms (42, 43).

Piezo1 in Immune Cells: Piezo1 plays a critical role in various immune cell types, including T cells, B cells, and NK cells. Studies have shown that the function of Piezo1 in T cells extends beyond the interaction between the T cell receptor (TCR) and major histocompatibility complex (MHC); it also plays a pivotal role in the formation of the immunological synapse (44). In NK cells, activation of Piezo1 enhances their cytotoxicity against tumor cells, promotes NK cell infiltration, and improves the efficacy of immunotherapy (19). Additionally, Piezo1 regulates the inflammatory responses and antigen-presenting functions of macrophages and DCs (24), both of which are key determinants of the immune landscape within the TME (44).

Piezo1 in Stromal Cells: Stromal cells, particularly fibroblasts and smooth muscle cells, serve as structural support within tissues and play critical roles in tumor progression and immune responses (40). The expression of Piezo1 in these cells is closely associated with tissue stiffness, mechanical tension, and pressure alterations within the TME (45). Through Piezo1-mediated mechanosensation, stromal cells adapt their functional behavior in response to mechanical cues, thereby influencing the plasticity of the TME and contributing to immune evasion by tumors.

## 3 The role of Piezo1 in the immune system

As a core member of mechanosensitive ion channels, Piezo1 plays a pivotal role in immune regulation by sensing mechanical cues from the microenvironment, thereby orchestrating a complex network spanning both innate and adaptive immunity. Its functional mechanisms involve not only the activation and modulation of immune cell function but also the dynamic balance between inflammatory responses and immune tolerance, ultimately shaping the overall landscape of the host immune response.

### 3.1 Piezo1 and immune cell activation

As a key mechanosensitive ion channel, Piezo1 plays a pivotal role in the activation, differentiation, and functional regulation of immune cells. Piezo1 is highly expressed in human T cells, where Ca²^+^ influx mediated by Piezo1 serves as a critical factor enhancing TCR signaling under mechanical stimulation. For instance, fluid shear stress can potentiate T cell activation via Piezo1, whereas pharmacological inhibition or genetic deletion of Piezo1 significantly attenuates mechanical force-induced ZAP70 phosphorylation and cytokine expression (16, 46). In B lymphocytes, Piezo1 participates in the early signal transduction triggered by membrane-bound antigens. Piezo1 enables B cells to sense mechanical cues associated with antigen presentation, thereby initiating B cell receptor (BCR) signaling cascades. Studies have shown that Piezo1 deficiency impairs B cell responses to immobilized antigens, while responses to soluble antigens remain largely unaffected. This suggests that Piezo1-mediated Ca²^+^ influx is essential for B cells to recognize surface-presented antigens and achieve full activation (18). In macrophages, the high expression of Piezo1 mRNA underscores its essential role in immune activation. Upon activation, Piezo1 facilitates Ca²^+^ influx and triggers multiple signaling pathways, promoting phagocytosis and regulating the secretion of proinflammatory cytokines (33, 47). Although conditional deletion of Piezo1 does not impair macrophage development, it significantly reduces their phagocytic capacity and reactive oxygen species (ROS) production during pathogen clearance (20). Moreover, functional expression of Piezo1 has also been detected in eosinophils. Activation of Piezo1—such as with the specific agonist Yoda1—induces robust Ca²^+^ influx, and further studies indicate that Piezo1 signaling modulates the expression and secretion of both pro- and anti-inflammatory cytokines in these cells (48). Collectively, these findings demonstrate that Piezo1 is ubiquitously expressed across both innate and adaptive immune cells, functioning as a mechanical sensor that converts extracellular physical stimuli into intracellular signaling. This positions Piezo1 as a critical regulator of immune cell activation, particularly in response to mechanical cues within the microenvironment.

### 3.2 Regulatory role of Piezo1 in immune responses

Piezo1 not only initiates immune cell activation but also fine-tunes the intensity and dynamics of immune responses by modulating the amplitude and duration of Ca²^+^ signaling. Recent studies have demonstrated that Piezo1 plays a critical regulatory role in macrophage activation, inflammatory responses, and functional polarization. Toll-like receptor (TLR) signaling pathways are pivotal in macrophage-mediated antimicrobial immunity. In macrophages, Toll-like receptor 4 (TLR4) interacts with the Piezo1 protein. Upon bacterial infection or lipopolysaccharide (LPS) stimulation, activation of Piezo1 induces Ca²^+^ influx, which subsequently triggers the phosphorylation of calcium/calmodulin-dependent protein kinase II (CaMKII) and the Hippo pathway kinases MST1 and MST2 (mammalian STE20-like protein kinases 1 and 2). This cascade leads to activation of the Rac signaling pathway, which regulates mitochondrial ROS production, thereby enhancing the phagocytic capacity and antimicrobial activity of macrophages (33). Furthermore, Piezo1-mediated Ca²^+^ influx activates the CaMKII-MST1/2-Rac signaling axis, reinforcing host immune responses by promoting pathogen clearance through macrophage phagocytosis (33), This signaling axis also prolongs the inflammatory response, contributing to a more robust and sustained immune reaction. In addition, studies have indicated that bone marrow-derived cells can sense cyclic hydrostatic pressure via Piezo1, which initiates inflammatory responses in monocytes and their macrophage derivatives *in vitro*. This mechanotransduction activates c-JUN and upregulates the transcription of endothelin-1 (EDN1), which in turn stabilizes hypoxia-inducible factor 1α (HIF1α). This cascade maintains elevated expression levels of pro-inflammatory mediators such as IL-1β, TNF-α, CXCL10, and PGE_2_, thereby prolonging the inflammatory response (18, 49). Consequently, mechanical stimuli, through Piezo1, drive macrophages to generate stronger and more persistent inflammatory signals. Collectively, Piezo1 plays a crucial role in amplifying innate immune responses by regulating the intensity and duration of macrophage activation upon stimulation.

In macrophages, Piezo1, as a mechanosensitive Ca²^+^ channel, responds to external mechanical cues such as matrix stiffness and shear stress by triggering intracellular Ca²^+^ influx, which subsequently activates a cascade of calcium-dependent signaling pathways. First, the rise in Ca²^+^ promotes calmodulin-mediated activation of calpain, which then cleaves and inhibits negative regulators of HIF-1α degradation, thereby stabilizing HIF-1α protein expression and enhancing its nuclear translocation (50). Meanwhile, Ca²^+^ also facilitates activation of the MAPK pathway, leading to phosphorylation of AP-1 and indirectly upregulating HIF-1α transcription (47). Through this dual mechanism—enhancing both protein stability and gene expression—Piezo1 promotes the expression of downstream proinflammatory mediators such as inducible nitric-oxide synthase (iNOS), IL-6, and TNF-α, thereby driving macrophage polarization toward the M1 phenotype (49, 51, 52). Conversely, loss or inactivation of Piezo1 leads to insufficient Ca²^+^ signaling, limited HIF-1α expression, and suppression of proinflammatory pathways, promoting polarization toward the anti-inflammatory M2 phenotype. These findings indicate that Piezo1 regulates macrophage fate through a mechanotransduction–Ca²^+^ signaling–metabolic-transcription axis, playing a key role in the functional reprogramming of TAMs.

Piezo1 also contributes to the optimization of immune responses by modulating the efferocytic capacity of macrophages. Tissue stiffness has been shown to influence the clearance function of macrophages, and activation of Piezo1 significantly enhances this capacity. Specifically, Piezo1 activation promotes lysosomal acidification during the phagocytic process, thereby improving the degradation efficiency of engulfed materials. Concurrently, it upregulates the expression of anti-inflammatory genes, contributing to the amelioration of the local immune microenvironment. This mechanism not only accelerates the resolution of inflammation but also facilitates the reversal of fibrotic tissue remodeling (27).

In addition to its regulatory role in macrophages, Piezo1 also responds to mechanical cues such as membrane ruffling induced by bacterial infection, highlighting its broader involvement in immune responses. During bacterial invasion, Piezo1 mediates Ca²^+^ influx, which subsequently triggers ATP release and initiates immune responses. This not only enhances host immune defense but also activates protective gene expression programs that further potentiate the immune response (53).

In summary, Piezo1 amplifies innate immune responses and enhances their efficacy by regulating macrophage activation, the duration of inflammatory responses, polarization states, and phagocytic function. Through the combined action of Ca²^+^ signaling and metabolic-transcriptional pathways, it promotes polarization toward the M1 phenotype and augments proinflammatory responses. Additionally, Piezo1 enhances the phagocytic capacity of macrophages, thereby improving the local immune microenvironment, accelerating inflammation resolution, and facilitating fibrosis reversal.

### 3.3 The role of Piezo1 in maintaining immune homeostasis

As a mechanosensitive ion channel, Piezo1 plays a multifaceted regulatory role in maintaining immune homeostasis. It precisely modulates the balance of immune responses by influencing immune cell differentiation, metabolic reprogramming, and mechanisms of immune tolerance.

**Figure 3:**
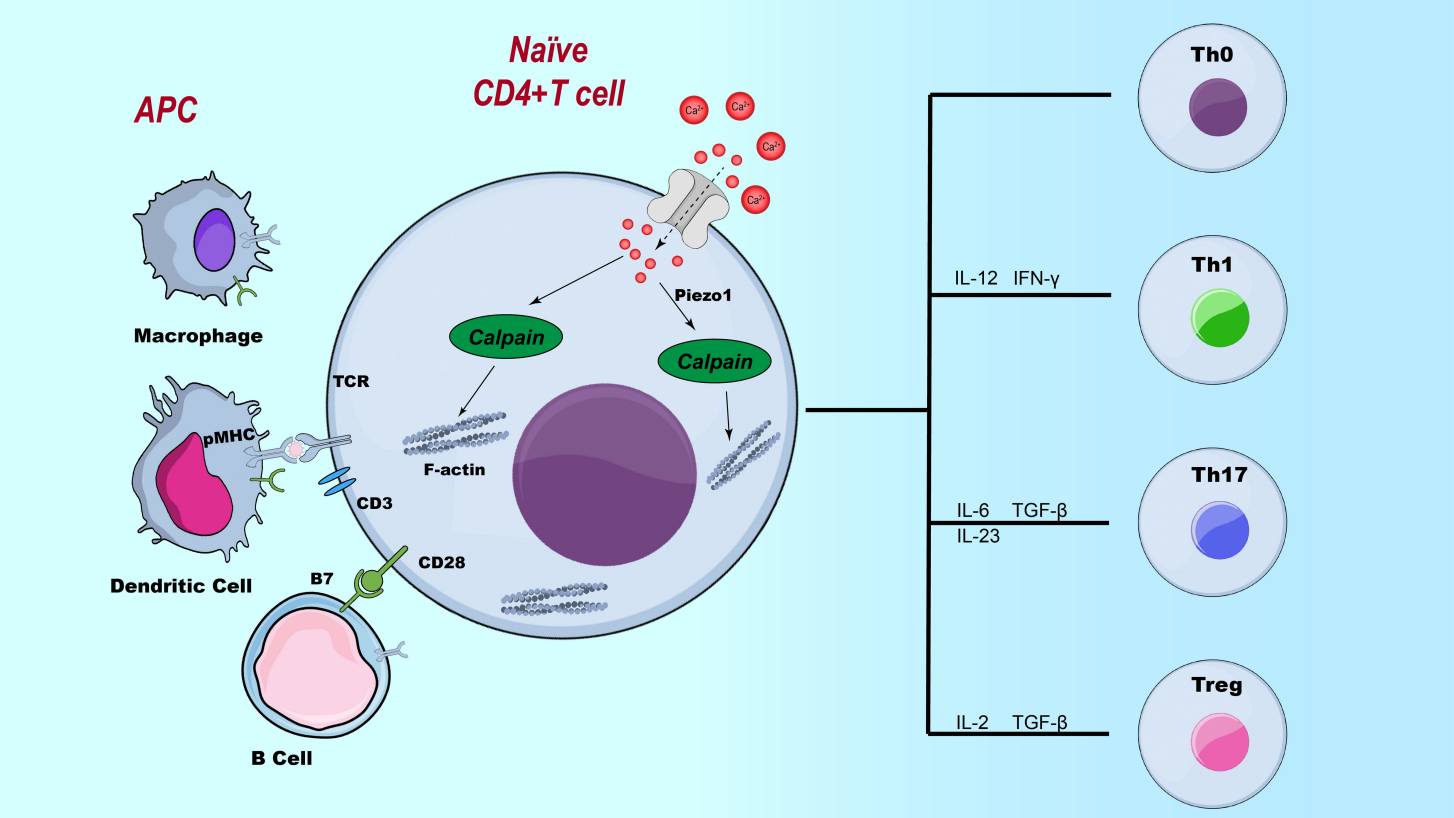
The role of Piezo1 in CD4^+^ T cell subset polarization. Illustration of the regulatory role of Piezo1 in T cell differentiation. This figure depicts the influence of Piezo1 signaling on the differentiation of various T cell subsets, including Th1, Th2, Th17, and Treg cells, highlighting its potential impact on immune responses.

During T cell differentiation, Piezo1 plays a critical role in maintaining immune homeostasis and preventing aberrant inflammatory responses by regulating the balance between pro-inflammatory Th1/Th17 cells and immunosuppressive Treg cells. On one hand, Piezo1 senses mechanical stimuli and suppresses the differentiation of Th1 and Th17 cells, thereby promoting immune tolerance and preventing the development of autoimmune diseases. CD4^+^ T cells deficient in Piezo1 exhibit abnormal secretion of IFN-γ and IL-17, leading to markedly enhanced inflammatory responses (13, 54) (As shown in **Figure 3**). On the other hand, although Piezo1 is necessary for the polarization of Th1 and Th17 cells, its deletion results in increased TGF-β signaling activity and an expanded Treg population, indicating that Piezo1 also plays a vital role in selectively restraining Treg cell function (21, 55). This regulatory mechanism helps prevent immune tolerance dysregulation due to excessive Treg activation while preserving the proper function of effector T cells during immune responses. In addition, Piezo1 modulates T cell function by integrating mechanical cues with metabolic reprogramming. Studies have shown that loss of Piezo1 leads to mitochondrial metabolic reprogramming, characterized by downregulation of sirtuin 3 (SIRT3) and suppression of oxidative phosphorylation (OXPHOS), resulting in the accumulation of ROS. This, in turn, promotes interleukin-9 (IL-9) expression via downstream HIF-1α signaling, thereby driving T helper 9 (Th9) cell lineage commitment. This process highlights the critical role of Piezo1 as a mechanotransduction–metabolic coupling hub (56). Therefore, Piezo1 is not only essential for maintaining immune tolerance but also influences the persistence and magnitude of immune responses through the regulation of T cell metabolism.

In the context of immune evasion, Piezo1 exerts multilayered and dual regulatory functions. On one hand, within the innate immune system, high expression of Piezo1 in macrophages and neutrophils facilitates the formation of neutrophil extracellular traps (NETs). Although NETs serve as an antimicrobial defense mechanism, their excessive release can create physical barriers that shield certain pathogens and tumor cells from immune recognition and clearance, thereby contributing to immune evasion to some extent (55). his highlights Piezo1 as a key regulatory factor in the immune escape process. On the other hand, Piezo1 modulates T cell migratory capacity by sensing local mechanical signals, thereby affecting the efficiency of immune surveillance. Piezo1 is predominantly localized at the leading edge of migrating T cells, where its recruitment is dependent on the formation of focal adhesions (FA) and is further enhanced by chemokine receptor activation. Upon activation, Piezo1 induces Ca²^+^ influx, which subsequently activates the calpain signaling pathway. This leads to the polarized clustering of the integrin LFA-1 (CD11a/CD18) at the migration front, thereby enhancing T cell adhesion to and migration toward target tissues (57).

Piezo1 is also closely associated with the regulation of inflammatory responses. Upon activation, Piezo1 induces intracellular Ca²^+^ influx, which subsequently activates the calcium-dependent potassium channel KCNN4, leading to potassium efflux and ultimately promoting the activation of the NLRP3 inflammasome (58). Excessive or uncontrolled inflammasome activation can trigger chronic inflammation, resulting in tissue damage and cell death (59). These findings suggest that Piezo1 may contribute to tumor initiation and progression by modulating chronic inflammatory responses within the TME.

In summary, Piezo1 is a critical component of the immune homeostasis regulatory network, exhibiting pronounced cell type–specific functions and microenvironment-dependent activity. Therapeutic strategies targeting Piezo1 must therefore be precisely tailored to the specific pathological context, offering significant potential in the treatment of autoimmune diseases and cancer immunotherapy.

## 4 The role of Piezo1 in tumor initiation and progression

### 4.1 Mechanosensitivity of tumor cells

Mechanosensitivity plays a critical role in tumor development and metastasis. The mechanosensitivity of tumor cells refers to their ability to perceive and respond to mechanical stimuli within the microenvironment. This involves the sensing, transmission, and transduction of mechanical signals, ultimately regulating key tumor cell behaviors such as proliferation, migration, invasion, drug resistance, and therapeutic responsiveness (60–62). Tumor cells can adapt to the mechanical stress of their surroundings by modulating their mechanical properties, including cellular stiffness, morphology, and adhesion, thereby promoting tumor growth and dissemination (63). Thus, a deeper understanding of tumor cell mechanosensitivity not only elucidates mechanisms underlying tumor progression but also offers novel strategies and targets for cancer diagnosis and therapy (64). As a pivotal mechanosensitive ion channel, Piezo1 plays a central role in tumor progression. Although the functional roles of Piezo1 may vary across different cancer types—regulating specific processes such as proliferation, migration, invasion, and epithelial–mesenchymal transition (EMT)—it commonly promotes cancer cell metastasis and invasion by altering tumor cell mechanical behavior. These include sensing matrix stiffness, modulating cellular traction forces, enhancing angiogenic factor production, and facilitating ECM remodeling (65).

### 4.2 Differential roles of Piezo1 across distinct stages of tumor progression

**Figure 4:**
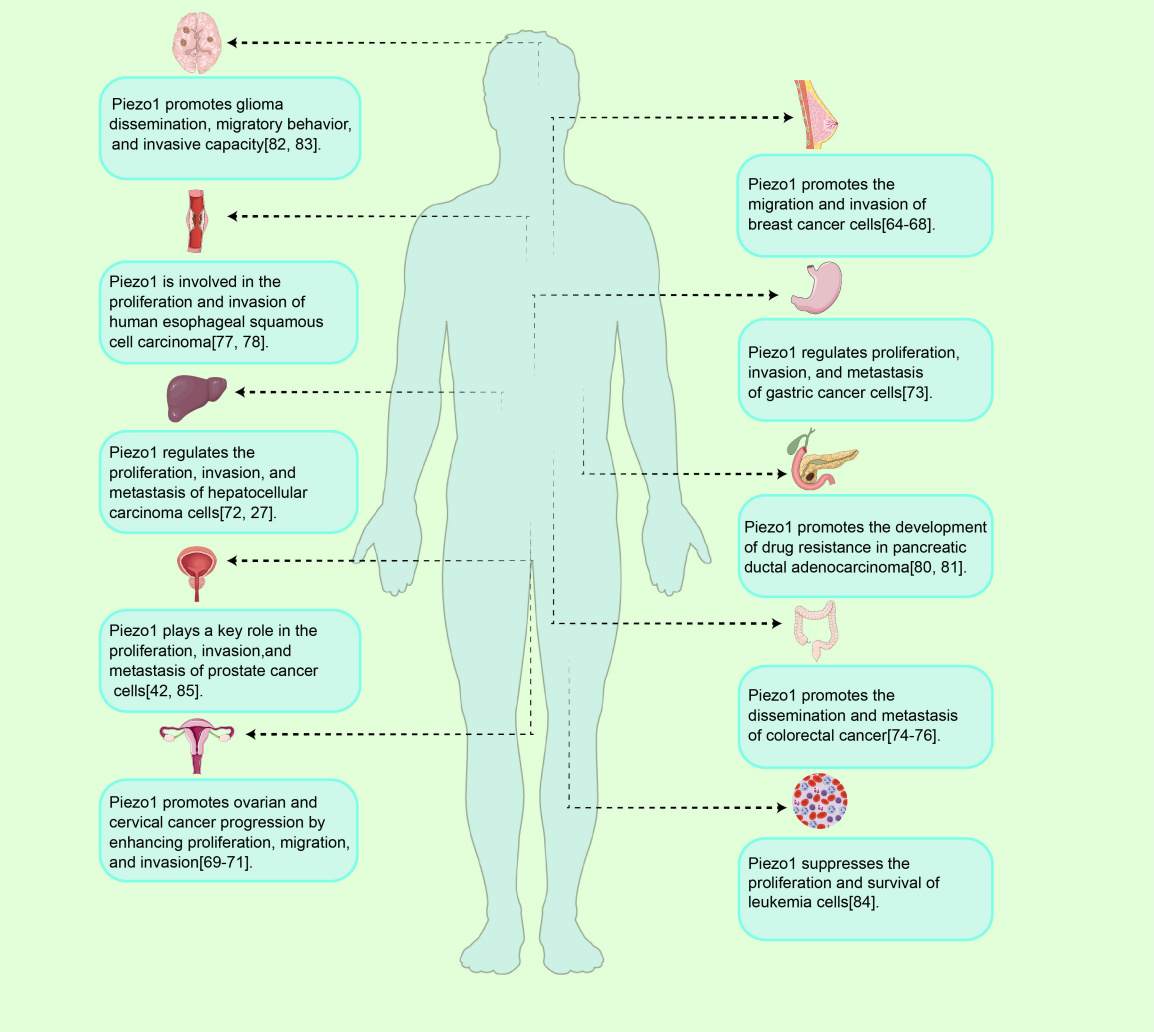
Piezo1 in tumor initiation and progression. Overview of Piezo1 functions in tumor progression. As a mechanosensitive ion channel, Piezo1 regulates tumor initiation and progression by modulating cellular processes such as proliferation, migration, invasion, and drug resistance, exhibiting both oncogenic and tumor-suppressive activities across various cancer types.

Piezo1 plays a significant role in various tumor cell types, although its functions differ depending on the cancer type(As shown in **Figure 4** and **Table 1**).

**Table 1:** Piezo1-associated signaling pathways and biological effects in various solid tumors.

Tumor type	Representative cancer type	Piezo1-associated signaling axis	Major biological effects
Digestive System Tumors	Gastric cancer	Activation of Rho GTPase family [Ref (66)]	Maintains spindle-shaped pseudopodia; enhances cell migration and invasion
	Colorectal cancer	Downregulation of MCU → Upregulation of HIF-1α/VEGF [Ref (67)]	Enhances mitochondrial adaptation, promotes metastasis and angiogenesis
	Esophageal squamous cell carcinoma	p53–Bax–Caspase-3 apoptotic axis [Ref (68)]	Inhibits apoptosis; promotes tumor cell survival
	Oral squamous cell carcinoma	YAP–Piezo1–Ca²^+^ positive feedback loop [Ref (69)]	Activates nuclear localization of YAP; enhances cell proliferation (↑Ki-67 expression)
	Pancreatic ductal adenocarcinoma	Piezo1–glycolytic metabolism axis [Ref (70)]	Promotes aerobic glycolysis; increases chemoresistance (e.g., to gemcitabine)
Gynecological Tumors	Cervical cancer	Piezo1–Ca²^+^–ATP positive feedback loop [Ref (71)]	Enhances pseudopodia formation and cell migration; drives tumor invasion
		Piezo1–ROS–Fe²^+^–GPX4 downregulation (induced by matrine) [Ref (72)]	Activates oxidative stress and ferroptosis; represents a druggable regulatory feature
	Ovarian cancer	Piezo1–Hippo/YAP–EMT axis [Ref (73)]	Promotes cell migration, EMT process, and distant metastasis
	Breast cancer	Piezo1–Ca²^+^–YAP1–EMP axis [Ref (74)]	In 2D, reduces adhesion and promotes migration; in 3D, enhances traction and invasion; induces E-cadherin↓, vimentin↑, SERPINE1↑ to drive EMP.
		Piezo1–Ezrin–Radixin–Moesin (ERM) inhibitory axis [Ref (75)]	Suppresses bleb-based amoeboid migration; regulates cortical stiffness and cytoskeletal architecture
		Piezo1–MMP signaling axis [Ref (63)]	Modulates expression of MMPs; affects ECM degradation and invasive potential
		Piezo1–GRHL3–RNF114/CREB–PD-1/TIM-3 axis [Ref (12) (76)]	Destabilizes immune synapse in T cells; promotes T cell exhaustion and immune evasion
Hepatobiliary Tumors	Hepatocellular carcinoma	Piezo1–Rab5c–Smad2/3–TGF-β signaling cascade [Ref (77)]	Drives EMT; enhances invasion and metastasis
		Piezo1–Ca²^+^–MAPK–YAP axis [Ref (27)]	Activates JNK/p38/ERK signaling; enhances cell survival and proliferation
Genitourinary Tumors	Prostate cancer	Piezo1–Ca²^+^–Akt–mTOR/CDK4–Cyclin D1 signaling [Ref (42)]	Promotes cell cycle progression and migration; improves adaptation to shear stress
Central Nervous System Tumors	Glioblastoma	Piezo1–TRAIL sensitivity axis [Ref (78)]	Reverses TRAIL resistance upon mechanical/pharmacological activation; enhances apoptosis
Hematological Tumors	Myeloid leukemia	Piezo1–cell cycle–DNA damage response pathway [Ref (79)]	Blocks G1/S transition; activates extrinsic apoptosis; suppresses leukemia cell survival

Breast Cancer: In breast cancer, Piezo1 exerts little influence on cell proliferation or cell-cycle progression but acts as a pivotal mechanosensitive node that regulates migration and invasion. Functional studies reveal a force-field-dependent duality: in two-dimensional (unconfined) substrates, knockdown of Piezo1 (Piezo1-KD) lowers cell adhesion and cortical stiffness, thereby accelerating random migration; in contrast, under three-dimensional confinement—such as narrow microchannels or pores—the same Piezo1-KD markedly impairs translocation owing to insufficient traction, indicating that Piezo1 functions as a “mechanical switch” that dictates directional motility according to spatial constraints. On the invasive front, Piezo1-KD diminishes invadopodium formation and down-regulates matrix metalloproteinase (MMP) family proteins, collectively suppressing extracellular-matrix degradation and penetration (63).

Notably, changes in cell morphology alone can trigger Ca²^+^ influx via Piezo1, remodeling cortical actin and fostering epithelial–mesenchymal plasticity (EMP): the Piezo1 agonist Yoda1 intensifies the Ca²^+^–YAP1 axis, leading to E-cadherin down-regulation and concomitant up-regulation of vimentin and SERPINE1, thereby driving EMP-linked invasive phenotypes (74). Piezo1 also modulates dynamic FA, influencing cellular stiffness and contractility, and suppresses thrombin-induced bleb formation; when Piezo1 is active, phosphorylation of ezrin-radixin-moesin (ERM) proteins is impeded, hindering bleb-dependent amoeboid migration (75). Consequently, Piezo1 acts both as a “sensor” of external mechanical cues and as a “threshold setter” that determines pro- or anti-tumor outcomes, with its function governed by the degree of spatial confinement, signal strength, and the extent of cell-shape remodeling.

Cervical and Ovarian Cancer: As a mechanosensitive ion channel, Piezo1 behaves as a “context-dependent switch” in gynecologic malignancies: it can either drive invasion and metastasis or, under specific chemical or mechanical cues, convert mechanical inputs into ferroptotic death signals—underscoring its complexity at the mechano-metabolic nexus.

Histopathological analyses show markedly elevated Piezo1 expression in cervical carcinoma, particularly in lymph-node–positive cases. Upon activation, Piezo1 triggers Ca²^+^ influx and massive extracellular ATP release, thereby enhancing pseudopodium formation, migration, and invasion; conversely, Piezo1 silencing or blockade sharply suppresses these phenotypes, whereas the selective agonist Yoda1 can “re-ignite” the invasive program—implicating a Piezo1–ATP–positive-feedback loop as a key mechanical driver of cervical-cancer metastasis (71). A separate study demonstrated that the phytochemical matrine up-regulates Piezo1, induces robust Ca²^+^ influx, and consequently promotes ROS and Fe²^+^ accumulation together with GPX4 down-regulation, culminating in canonical ferroptosis. This effect is independent of the classical iron-metabolism components transferrin receptor (TFR) and cystine/glutamate transporter (xCT) and is completely reversed by Piezo1-siRNA (72). Thus, Piezo1 can serve as a druggable gatekeeper that converts mechanical or chemical stimuli into lethal oxidative stress. Piezo1 is also highly expressed and mechanosensitive in ovarian carcinoma. When the mechanical landscape of the TME changes, Piezo1 becomes activated and specifically engages the Hippo/YAP axis, driving EMT and enhancing the migratory and metastatic potential of A-1847 and 3AO cells. Functional assays show that Piezo1-shRNA markedly slows subcutaneous tumor growth in nude mice, suppresses scratch-wound migration, and reduces pulmonary metastatic foci, whereas Yoda1 restores YAP activation and cell motility. Hence, Piezo1 promotes ovarian-cancer growth and dissemination via a mechanical–Hippo/YAP–EMT pathway, and its inhibition or blockade represents a promising anti-metastatic strategy (73).

In sum, Piezo1 can act both as a pro-invasive mechanosensor and, under particular compound or force inputs, as a ferroptosis gatekeeper. Its functional outcome depends on stimulus type, magnitude, and temporal window. Precisely toggling Piezo1 between “anti-metastatic” and “pro-killing” modes remains unexplored; future work must systematically define dose-time parameters and develop tissue-specific delivery systems to ascertain its true clinical value while minimizing off-target risks.

Hepatocellular carcinoma (HCC): In HCC, Piezo1 expression is markedly up-regulated and correlates positively with tumor aggressiveness and poor prognosis, suggesting that Piezo1 is a key driver of disease progression. One pivotal mechanism involves transforming growth factor-β (TGF-β) signaling, a canonical pathway that promotes tumor proliferation and metastasis. Recent work demonstrates that Piezo1 recruits Ras-related protein Rab5c (Rab5c), thereby facilitating phosphorylation of SMAD family member 2/3 (SMAD2/3) and activating the classical TGF-β cascade to accelerate HCC progression and EMT (77).

Piezo1 is also expressed in the HepG2 cell line, where it occupies the apex of a Ca²^+^-MAPK–YAP axis: activation of Piezo1 by the agonist Yoda1 induces Ca²^+^ influx and, in a time- and dose-dependent manner, phosphorylates c-Jun N-terminal kinase (JNK), p38, and extracellular signal-regulated kinase (ERK), thereby fully engaging MAPK signaling. Activated MAPK subsequently drives YAP nuclear translocation and downstream gene expression, enhancing proliferation and migration. Conversely, Piezo1 depletion suppresses MAPK–YAP activation, lowers calpain activity, and increases apoptosis; in Piezo1-haploinsufficient mice, HCC growth is likewise impeded. Collectively, the Piezo1/MAPK/YAP cascade is a critical control point for HCC cell survival and invasion, underscoring the therapeutic potential of Piezo1 as a diagnostic and interventional target (27). Pharmacologic disruption of the Piezo1–MAPK–YAP signaling circuit may therefore curb HCC progression and offer a novel clinical strategy.

Digestive-system tumors: In digestive and associated glandular malignancies, mechanosensitive ion channel Piezo1 is broadly upregulated and significantly associated with invasive phenotypes and poor prognosis; however, its oncogenic mechanisms show marked tissue-specific downstream pathway divergence, reflecting the biological feature of “homologous channel–divergent outputs.” In gastric cancer, the expression of Piezo1 is significantly elevated in most cell lines and primary samples. Piezo1 activates the Rho GTPase family to maintain spindle/fibroblast-like pseudopodia, thereby enhancing cell migration and invasion, and its high expression is associated with worse disease-specific survival (66). In colorectal cancer, Piezo1 upregulation correlates with poor prognosis. Mechanical stress activates the Piezo1 signaling pathway, leading to downregulation of mitochondrial calcium uniporter (MCU), upregulation of HIF-1α and vascular endothelial growth factor (VEGF), and reduced mitochondrial membrane potential generation, which together promote colorectal cancer metastasis (67, 80, 81). In esophageal squamous cell carcinoma (ESCC), Piezo1 upregulation activates the p53–Bax–Caspase-3 apoptosis pathway to resist apoptosis, while knockdown of Piezo1 reactivates this pathway and blocks the G0/G1→S transition, exhibiting a function as an “anti-apoptotic switch” (68, 82). In oral squamous cell carcinoma (OSCC), mechanical changes in the TME activate the Hippo–YAP signaling pathway to promote proliferation. Loss-of-function studies and patient immunohistochemistry show that YAP nuclear localization is closely associated with OSCC proliferation. Transcriptomic analysis further identified Piezo1 as a direct transcriptional target of YAP upon upregulation, YAP induces Piezo1, leading to Ca²^+^ influx and amplification of proliferative signaling, while inhibition of YAP or knockdown of Piezo1 significantly suppresses tumor cell growth in 2D, 3D, and suspension cultures. In clinical samples, regions of YAP nuclear/cytoplasmic overexpression co-localize with high levels of Piezo1 and the proliferation marker Ki-67, while non-tumor tissues lack this signaling axis. Thus, the YAP–Piezo1–Ca²^+^ cascade constitutes the core growth-driving module of OSCC, linking external mechanical stimulation to internal proliferation programs (69). Pancreatic ductal adenocarcinoma (PDAC) is a highly aggressive solid tumor characterized by significantly increased tissue stiffness, which has been shown to be closely associated with tumor progression (83). Under high-stiffness stimulation, the expression of Piezo1 is significantly elevated in PDAC cells. Piezo1 promotes chemoresistance to gemcitabine by activating the glycolytic metabolic pathway. Notably, selective inhibition of Piezo1 not only effectively suppresses glycolysis but also partially restores chemosensitivity, offering a promising therapeutic strategy for overcoming PDAC chemoresistance (70). Although current evidence supports Piezo1 as a mechanosensory oncogenic hub in various gastrointestinal tumors, the downstream effectors involved (Rho, MCU, p53, YAP, metabolic enzymes) differ significantly between tissues, indicating that its function is co-shaped by the mechanical and metabolic microenvironment. Most existing data come from single-cell line or xenograft models, lacking orthotopic chronic models and verification of immune–mechanical interactions. Future research should focus on *in situ* mechanical imaging, multi-omics analysis, and the development of controllable Piezo1 modulators to define its true therapeutic window.

Other malignancies: Beyond digestive system tumors, Piezo1 also demonstrates high plasticity as a “mechanotransduction–signaling hub” in central nervous system, hematological, and prostate malignancies. It can serve not only as a prognostic marker but also as a functional regulator of proliferation or chemosensitivity. In glioblastoma (GBM), high Piezo1 expression is associated with poor prognosis. Chemical or mechanical activation of Piezo1 enhances tumor cell sensitivity to tumor necrosis factor–related apoptosis-inducing ligand (TRAIL)-induced apoptosis, thereby reversing resistance to temozolomide (TMZ) (78, 84), offering a highly specific and minimally invasive therapeutic strategy for this lethal brain tumor. In myeloid leukemia cell lines, downregulation of PIEZO1 significantly impairs cell proliferation and survival. PIEZO1 knockdown blocks normal cell cycle progression at the G0/G1 phase, disrupts the DNA damage response, and significantly increases cell death through activation of the extrinsic apoptotic pathway (79). In prostate cancer cell lines and malignant human tissue samples, Piezo1 expression is markedly elevated. This channel mediates Ca²^+^ influx to activate the protein kinase B (Akt)–mechanistic target of rapamycin (mTOR) axis and the CDK4/cyclin D1 complex, thereby promoting proliferation and migration. Piezo1 silencing inhibits Ca²^+^–Akt–mTOR signaling, arrests cells in G0/G1, and attenuates migration; it also blocks the wall shear stress–YAP/transcriptional co-activator with PDZ-binding motif (TAZ) pathway, thereby inhibiting PC3 cell growth and metastasis *in vivo (* 42, 85).

In summary, Piezo1 is not merely a “pro-oncogenic” or “tumor-suppressive” factor, but rather a “biomechanical–metabolic switching threshold.” Upon activation, the magnitude and duration of Ca²^+^ signals, together with ECM stiffness, oxygen metabolism, and other contextual variables, jointly determine whether downstream signaling promotes proliferation/invasion or apoptosis/ferroptosis. Only when external resistance or matrix rigidity surpasses a defined “mechanical threshold,” and downstream modules (such as Rho, Hippo–YAP, mitogen-activated protein kinase[MAPK], and metabolic enzymes) are correctly coupled, can Piezo1–Ca²^+^ signals shift from a “relaxed mode” to a “tensional mode,” thereby driving tumor cell migration, drug resistance, and metastasis. Conversely, decoupling or pharmacologic redirection of this axis (e.g., via matrine) may convert the same signals into ROS imbalance and ferroptotic death. To achieve effective clinical outcomes such as “anti-metastasis” or “pro-death,” it is imperative to: (1) quantify the mechanical–Ca²^+^ activation threshold and map predictive dose–time curves; (2) develop tissue-specific Piezo1 modulators to prevent off-target effects; and (3) decode the tri-modal interaction among immunity, mechanics, and metabolism in orthotopic or humanized models. Only by clarifying these critical variables can Piezo1 be transformed from a “descriptive biomarker” into a programmable therapeutic switch, offering controllable and precise mechanometabolic interventions for highly aggressive and drug-resistant tumors.

### 4.3 Regulatory role of Piezo1 in the tumor microenvironment

**Figure 5:**
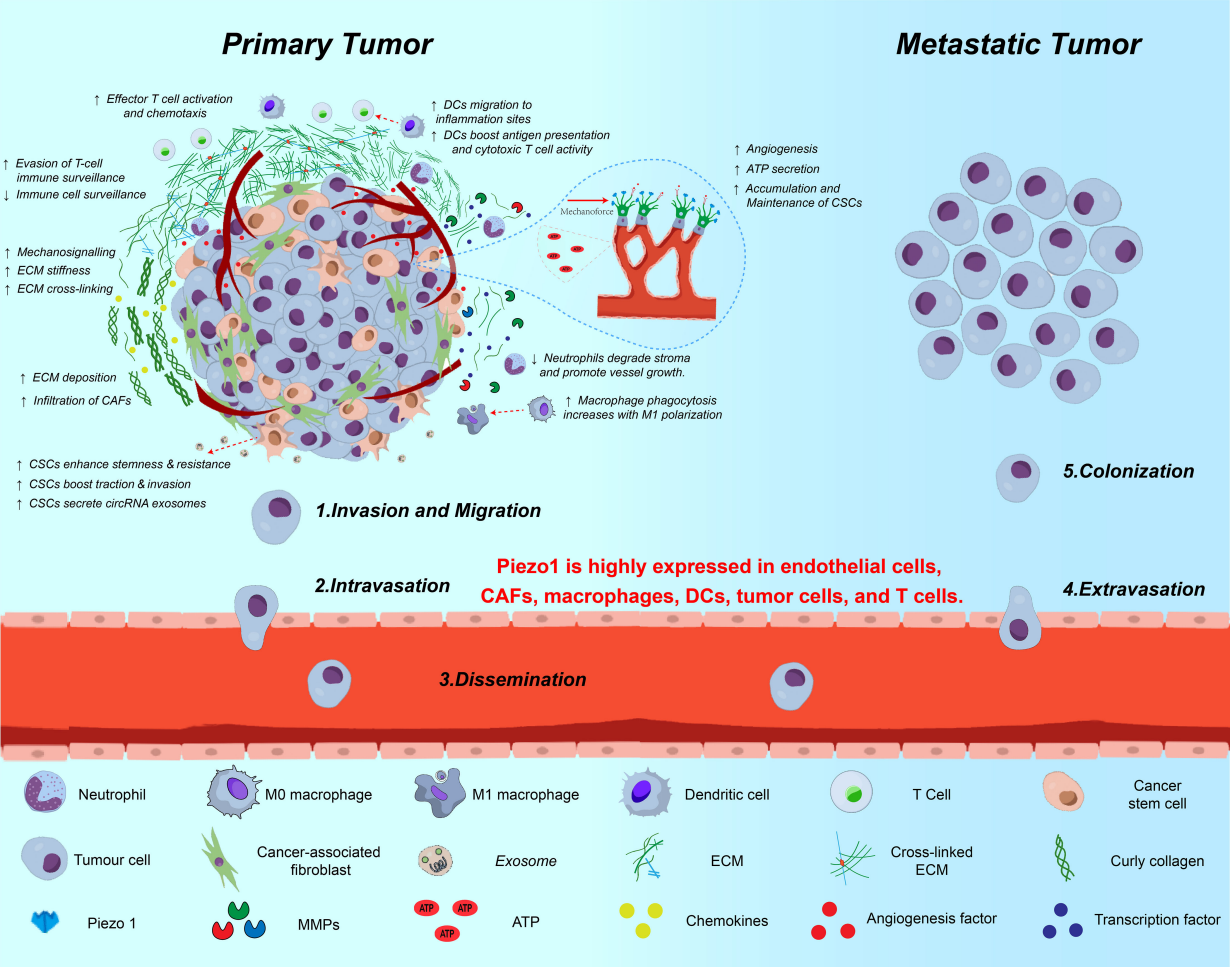
Multifaceted roles of Piezo1 in tumor progression and metastasis. This schematic illustrates the involvement of Piezo1 across various stages of cancer progression—from local invasion to distant metastasis. In primary tumors, Piezo1 senses matrix stiffness and transduces mechanical cues to promote ECM remodeling, CSC stemness, EMT, and immune suppression. It is highly expressed in tumor cells, endothelial cells, CAFs, DCs, macrophages, and T cells. Piezo1-mediated Ca²^+^ influx enhances invadopodia formation, exosome secretion, ATP release, and MMP activation, thereby facilitating migration, intravasation, and ECM degradation. During dissemination and colonization, Piezo1 contributes to vascular remodeling, neovascularization, and immune escape, ultimately promoting metastatic niche formation and CSC maintenance.

The TME is a dynamic ecosystem shaped by innate inflammatory stimuli (such as infectious factors, hypoxia, and nutrient competition) and mechanical signals (such as matrix stiffness and fluid shear stress). Within this environment, interactions among immune cells (such as T cells and DCs), stromal cells (such as CAFs), and the ECM drive tumor proliferation, metastasis, and immune evasion (13, 86). As a central hub for mechanical signal transduction in the TME, Piezo1 integrates mechanical and biochemical signals to regulate several key aspects of tumor progression(As shown in **Figure 5**).

#### 4.3.1 Piezo1-mediated cell–matrix interactions and tumor cell migration

During tumor progression, the stiffness of the ECM increases continuously, a process primarily driven by CAF-mediated collagen remodeling. The resulting mechanical tension initially activates Piezo1, which is highly expressed at both intracellular and extracellular domains, positioning it as a mechanical hub for tumor cell–ECM communication (86). In geometrically confined microenvironments, Piezo1-mediated Ca²^+^ influx and traction force redistribution not only reshape cellular morphology but also globally enhance cell–matrix adhesion, thereby promoting more efficient migration and invasion (87).

In high-stiffness tumors such as glioblastoma, excessive activation of Piezo1 synergizes with the integrin–focal adhesion kinase (FAK) signaling axis, forming a positive feedback loop—”increased stiffness → enhanced Piezo1 currents → FAK activation → collagen redeposition → further stiffness elevation”—which correlates with reduced patient survival (84). Similarly, the Piezo1–YAP axis in CAFs is activated under high mechanical tension, stimulating the secretion of collagen, fibronectin (FN), TGF-β, and interleukin-6 (IL-6), which in turn promotes ECM fibrosis and further enhances cancer cell invasion. The connective tissue growth factor (CTGF)–YAP1 axis in gastric cancer exemplifies such “bidirectional coupling” (88, 89).

Notably, Piezo1 is not solely a “mechanical reinforcer.” Its Ca²^+^ signal can also induce matrix metalloproteinases (MMP)-1/-10 expression, facilitating local ECM degradation and reducing adhesion, thereby aiding tumor spheroid detachment—an essential mechanism for ovarian cancer peritoneal dissemination (90). In breast and colorectal cancers, Piezo1 upregulation is associated with decreased E-cadherin and increased N-cadherin and Snail expression, indicating its regulatory role in EMT and adhesion remodeling (74, 91).

At the cancer stem cell level, Piezo1 is highly expressed in glioblastoma CSCs (GBM-CSCs) compared to non-CSCs, correlating with increased FA and contractile force. The downstream RhoA–actomyosin contraction module allows CSCs to maximize invasive efficiency under confined geometries (41, 92, 93). Simultaneously, Piezo1 activation promotes the release of extracellular vesicles enriched with circular RNAs (circRNAs), enabling mechanical communication with neighboring cells and expanding its biomechanical influence radius (94).

On metabolic and immunological fronts, Piezo1-triggered ATP release can shift cancer cells toward a dual-activated state characterized by high oxidative phosphorylation (OXPHOS) and glycolysis (95). Additionally, the shear stress–Piezo1–ATP axis may contribute to neovascularization and affect CAF/immune cell infiltration, suggesting a potential immunosuppressive role (12, 96).

Taken together, Piezo1 plays a pivotal role in modulating TME dynamics. However, its pleiotropic functions and complex regulation—modulated by ECM stiffness, cell type, and microenvironmental cues—pose significant challenges for mechanistic study. While Piezo1 is considered a promising therapeutic target, its activation involves multiple biological pathways. Overinhibition could provoke off-target effects, highlighting the necessity of developing precisely controlled therapeutic strategies for modulating Piezo1 activity.

#### 4.3.2 Piezo1-mediated mechanotransduction and tumor angiogenesis

Within the TME, angiogenesis is not only a key process in structural remodeling but also provides essential material and pathways for tumor growth and metastasis. In recent years, Piezo has garnered increasing attention for its role in regulating angiogenesis. In vascular endothelial cells, Piezo1 functions as a mechanical–biochemical signal transducer by sensing variations in shear stress and membrane tension. Specifically, leading tip endothelial cells use Piezo1 to detect fluid shear forces, which triggers Ca²^+^ influx and activates the Notch–Dll4 signaling axis, thereby regulating the fate of adjacent stalk cells and ensuring directional and orderly vascular sprouting (97, 98).

During later stages of blood flow stabilization, Piezo1 also maintains vascular tension through modulation of cellular tension, preventing excessive dilation or collapse and ensuring dynamic perfusion homeostasis (99). In various tumor types—including gliomas, gastric, breast, and liver cancers—Piezo1 is highly expressed. Upon activation, it facilitates Ca²^+^ influx, which inhibits the ubiquitination and degradation of HIF-1α, thereby stabilizing HIF-1α and enhancing the expression of pro-angiogenic factors such as VEGF, CXCL16, and IGFBP2, accelerating pathological angiogenesis (67, 91).

More complexly, Piezo1 mediates not only endothelial cell-autonomous responses but also intercellular communication. For instance, M2-type TAMs can secrete platelet-derived growth factor-BB (PDGF-BB) and bone morphogenetic protein-2 (BMP-2), enhancing the sensitivity of adjacent endothelial cells to Piezo1 stimulation and inducing expression of matrix metalloproteinases MMP-2 and MT1-MMP, thereby promoting basement membrane remodeling and neovessel formation (100). In addition, Piezo1 responds to wall shear stress and sphingosine-1-phosphate (S1P), both of which are pro-angiogenic stimuli. These signals activate Ca²^+^ influx through Piezo1 and stimulate MMP-2 and MT1-MMP expression, facilitating endothelial cell filopodia extension and lumen formation to further promote angiogenesis (101).

Functional studies confirm that silencing Piezo1 significantly impairs VEGF-induced migration and directional alignment of human umbilical vein endothelial cells (HUVECs), indicating that Piezo1 is critical for vascular morphogenesis (9, 102). Furthermore, Piezo1 may regulate gene expression patterns associated with the TME, thereby influencing the balance between epithelial cell proliferation and apoptosis, disrupting the integrity of tissue barriers, and promoting the aberrant reorganization of tumor vasculature (103).

Although Piezo1 has been identified as a key sensor and effector in tumor angiogenesis, whether it acts in conjunction with tumor-type-specific cofactors or exhibits dose- and time-dependent functional polarity remains unclear. Future research should aim to elucidate the mechano-metabolic-immunological interaction network mediated by Piezo1, with the goal of developing targeted and controllable strategies to inhibit pathological neovascularization and restore perfusion homeostasis.

#### 4.3.3 Piezo1-mediated immune cell interactions and the formation of an immunosuppressive microenvironment

Although tumors are continuously surveilled by the host immune system, they often reshape the tumor immune microenvironment (TIME) to gradually establish an immunosuppressive state that enables immune evasion. Monocytes/macrophages, T cells, and B cells collectively form the core of the TIME and play decisive roles in maintaining or disrupting immune homeostasis (104).

As a mechanosensitive ion channel broadly expressed on the membranes of various immune cells, Piezo1 plays a crucial role in the differentiation, activation, and functional regulation of immune cells. Recent evidence indicates that the role of Piezo1 has expanded from controlling “individual cellular behavior” to coordinating “multicellular interactions.” This is particularly prominent in T cells, macrophages, and other myeloid cells, where Piezo1-dependent functional reprogramming is especially evident.

In the DC–effector T cell axis, Piezo1 integrates mechanical cues and metabolic reprogramming into the antigen-presenting function of DCs. Upon activation, Piezo1 promotes Ca²^+^ influx, which upregulates TGF-β1 secretion via the Ca²^+^–calcineurin–NFAT pathway. Concurrently, it enhances glycolytic flux through the SIRT1–HIF-1α axis, skewing DC differentiation toward a “low-stimulatory” phenotype with reduced CD80/CD86 expression. This impairs the capacity of DCs to polarize CD4^+^ T cells toward Th1 while promoting Treg expansion (105).

Additionally, Piezo1 disrupts MHC-I antigen processing and reduces the stability of peptide–MHC complexes, weakening DC–CD8^+^ T cell interactions. Notably, under non-tumor or acute infection conditions, Piezo1 may enhance type I interferon production by DCs, thereby activating NK cells and cytotoxic T lymphocytes (CTLs) (106, 107). However, in solid tumors, persistent mechanical and inflammatory stimuli bias Piezo1 toward driving TGF-β–dependent immunosuppressive polarization, indicating its pronounced microenvironmental context dependence.

**Figure 6:**
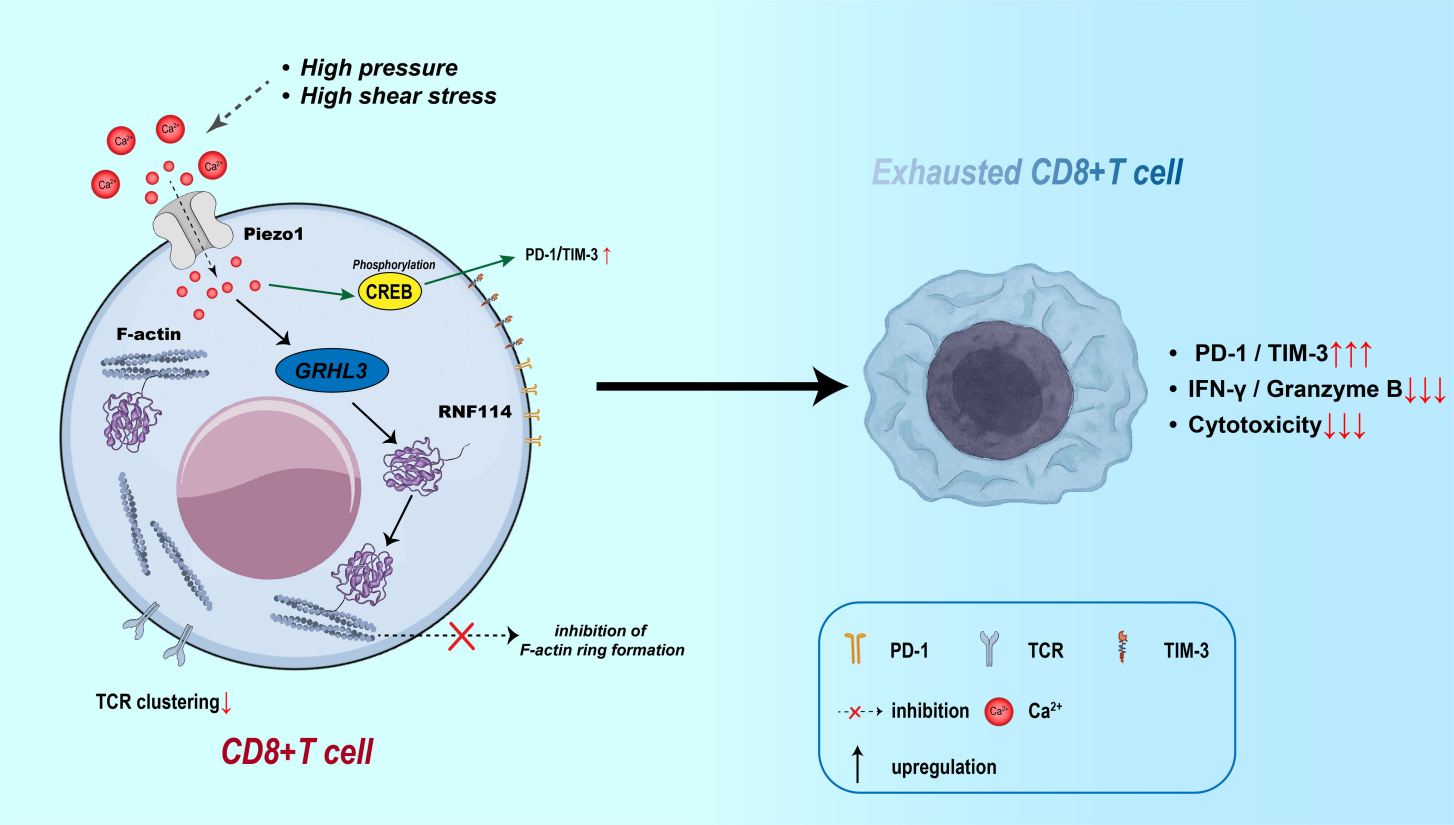
Piezo1-induced CD8^+^ T cell exhaustion via Ca²^+^ signaling and immune synapse disruption. Mechanical stress activates Piezo1 in CD8^+^ T cells, inducing Ca²^+^ influx and CREB phosphorylation, which upregulates PD-1 and TIM-3. Meanwhile, the GRHL3–RNF114 axis impairs F-actin ring formation and TCR clustering, disrupting the immune synapse. These effects contribute to CD8^+^ T cell exhaustion, marked by increased checkpoint expression and reduced cytotoxic function.

In the direct interaction between T cells and tumor cells, Piezo1 primarily modulates cytotoxicity by regulating immune-synapse structural stability and T-cell traction (57). Studies show that, under conditions of elevated membrane tension, Piezo1 activation promotes Ca²^+^ influx, which induces calpain activation and reorganizes the cortical F-actin network, thereby facilitating immune-synapse formation (17). However, within the TME, T cells chronically exposed to high pressure and shear forces experience sustained Piezo1 activation that ultimately destabilizes the immune synapse—evidenced by abnormal redistribution of peripheral adhesion proteins, diminished T-cell receptor (TCR) clustering, and consequent loss of cytotoxic function. Mechanistic analyses reveal that Piezo1 suppresses F-actin adhesion-belt assembly through the GRHL3–RNF114 signaling axis and, by enhancing CREB phosphorylation, up-regulates checkpoint molecules such as PD-1 and T-cell immunoglobulin and mucin-domain containing-3 (TIM-3), driving a state of T-cell exhaustion (12, 108) (As shown in **Figure 6**). In hormone-receptor–negative tumors—particularly triple-negative breast cancer—high Piezo1 expression correlates with reduced CD8^+^ T-cell infiltration, poor therapeutic response, and unfavorable prognosis (76). Pharmacologic inhibition of Piezo1 (e.g., with GsMTx4) can partly restore T-cell infiltration and boost effector function, highlighting the bidirectional regulatory role of Piezo1 in T-cell–tumor cell crosstalk.

TAMs interact with T cells in a manner that is strongly modulated by Piezo1. Macrophages—one of the most abundant immune populations in the TME—can adopt either an M1 (antitumor) or M2 (protumor) phenotype, and blocking M2 polarization while driving re-polarization from M2 to M1 is considered a promising therapeutic strategy (109). Studies show that, under increased tissue stiffness and matrix fibrosis—physical hallmarks of the TME—both the expression and activation of Piezo1 in macrophages rise sharply, promoting M2 polarization via a Ca²^+^-dependent dephosphorylation and nuclear translocation pathway involving YAP (110). Beyond their elevated secretion of immunosuppressive cytokines such as IL-10 and TGF-β, M2 macrophages up-regulate PD-L1 and bind PD-1 on T-cell surfaces, directly inducing T-cell exhaustion (52, 111). They also recruit Tregs through chemokines such as CCL22, thereby expanding the immune-tolerant network. Notably, several key steps in this process—YAP activation in TAMs, IL-10 production, and synthesis of Treg-attracting chemokines—are all governed by Piezo1 signaling (105, 112). As mentioned earlier, connective tissue growth factor (CTGF) secreted by CAFs can enhance YAP signaling in M2 TAMs via Piezo1, while TAMs in turn promote CAF activation and ECM remodeling, together forming a CAF–TAM–T-cell tri-cellular positive feedback loop that reinforces immunosuppression in the TME (113).

Finally, myeloid-derived suppressor cells (MDSCs)—another key immunosuppressive population within the TME—also exhibit a composite phenotype of enhanced proliferation, increased chemotaxis, and heightened suppressive activity under Piezo1 regulation (114). Elevated matrix stiffness or fluid-dynamic stress induces histone deacetylase 2 (HDAC2) via Piezo1, which epigenetically silences Rb1 and skews myeloid progenitors toward MDSC differentiation (115–117). Concurrently, the Piezo1–CXCL12/CXCR4 axis recruits myeloid cells (e.g., monocytes, MDSCs) and may indirectly impair CD8^+^ T-cell function by remodeling the tumor milieu (118, 119). Under sustained Piezo1 activation, MDSCs up-regulate immunosuppressive mediators such as arginase-1 (Arg1) and iNOS, leading to nutrient depletion and increased ROS production, which further suppress T-cell proliferation and function (120, 121). In chemoresistant tumor models—including pancreatic and liver cancers—the activation of the Piezo1–MDSC axis is a major contributor to immunotherapy failure (122).

In summary, Piezo1 plays multifaceted roles in the tumor immune microenvironment, promoting immune evasion by modulating immune-cell functions. It alters dendritic-cell antigen presentation, drives T-cell exhaustion, and directs macrophage polarization, thereby weakening antitumor responses while amplifying the suppressive actions of TAMs and MDSCs. The context-dependent effects of Piezo1 across immune subsets can favor either immune tolerance or activation. Although Piezo1 is an attractive therapeutic target, its bidirectional regulation and intricate interplay with the TME add layers of complexity. Future studies should elucidate tumor-specific mechanisms of Piezo1 and optimize its exploitation as an immunotherapeutic target.

## 5 Application of Piezo1 in personalized treatment

### 5.1 *In vivo* research progress toward clinical translation

Although extensive *in vitro* studies have revealed the critical role of Piezo1 in cancer initiation and progression, *in vivo* investigations provide more direct and practically relevant evidence for clinical translation. Currently, Piezo1 modulators have demonstrated certain biological activities in various tumor models, with research primarily focusing on the selective agonist Yoda1 and the non-specific inhibitor GsMTx4. Recent studies have shown that low-frequency ultrasound (LFS) can inhibit the malignant behavior of keloids by activating Piezo1 channels, and Yoda1 mimics this physical stimulus to exert antiproliferative effects, indicating its potential therapeutic value in hyperproliferative disorders (123).

Moreover, Yoda1 significantly suppresses EGF-induced macropinocytosis in Ras-transformed tumor cells, thereby interfering with exogenous amino acid uptake and inhibiting rapid tumor cell proliferation, providing a novel chemical strategy for cancer therapy (124). In melanoma models, Yoda1 has also been shown to inhibit tumorsphere formation, suggesting a potential application in targeting tumor stemness (125).

Notably, the biological effects of Piezo1 modulators may vary across different tumor types. For example, in cervical and pancreatic cancer models, Yoda1 promotes tumor growth and metastasis by inducing ATP release, activating the YAP/TAZ signaling pathway, and interacting with the TME (71, 126). In contrast, GsMTx4 suppresses EMT and reduces metastatic capacity in lung adenocarcinoma and ovarian cancer models by inhibiting the ROS/Wnt/β-catenin and Hippo/YAP pathways (73, 127). Additionally, this inhibitor facilitates immune cell infiltration into the TME by modulating immune signaling axes (12).

These findings highlight the complexity of Piezo1-targeted modulation, with its biological effects being significantly influenced by tumor type, developmental stage, and microenvironmental context.

### 5.2 Development and optimization of novel Piezo1 agonists

Despite the aforementioned advances, applying Piezo1 modulators *in vivo* remains challenging. First, their biological effects are bidirectional: both Yoda1 and GsMTx4 can be “pro-tumor” or “anti-tumor,” depending on cancer type, stage, and microenvironmental context, complicating precise therapeutic prediction. Second, target specificity is limited. Members of the mechanosensitive channel family share high structural and functional similarity (128–130); their downstream signaling pathways exhibit extensive crosstalk (131), and their actions are dose-dependent (132), causing Yoda1 and GsMTx4 to elicit off-target effects that hinder precise therapy. Third, each drug has intrinsic shortcomings. Yoda1 has poor solubility and unfavorable pharmacokinetics with systemic delivery, leading to off-target issues (133), whereas GsMTx4 shows high immunogenicity and poor stability, compromising clinical safety and efficacy. Fourth, research remains at an early stage: most studies are confined to animal models, lacking systematic human data and comprehensive preclinical evaluation, preventing these modulators from readily advancing into clinical development.

To overcome these limitations, researchers have recently developed new Piezo1 agonists through structural modification and high-throughput screening. Yoda2, which introduces a 4-benzoic acid substituent, improves Piezo1 selectivity (134). A deuterated compound based on a (thiadiazol-2-yl)-pyrazine scaffold exhibits an EC_50_ of 2.21 μM—more than 20-fold greater potency than Yoda1 (135). Another novel agonist, Yaddle1 (EC_50_ = 0.40 μM), further enhances activity by incorporating a trifluoromethyl group (136). These next-generation agonists display superior selectivity and pharmacodynamics in animal studies, and several have completed preliminary *in vivo* distribution and toxicity assessments. Future work must validate durability and specificity across multiple cancer models in mice and integrate protein-structure insights to refine preclinical candidates for clinical translation.

### 5.3 Gene-intervention strategies: targeted regulatory potential of siRNA and CRISPR

In addition to small-molecule modulators, gene-level targeted intervention has been regarded as a key complementary approach for precisely regulating Piezo1 activity, offering new strategies for achieving cell-selective inhibition. siRNA and CRISPR/Cas9 are currently the two most actively studied techniques. siRNA can reduce Piezo1 expression by transiently degrading mRNA: in cervical cancer models, Piezo1-siRNA significantly inhibits tumor cell invasion and migration (71); in hepatocellular carcinoma models, siRNA further suppresses proliferation, enhances autophagy, and synergistically increases doxorubicin (DOX) sensitivity by inhibiting the PI3K/AKT/mTOR signaling pathway. Combined use with Jianpi Huayu prescription (JPHY) further reduces tumor burden without evident hepatotoxicity or nephrotoxicity (137). However, siRNA still carries the risk of off-target effects due to partial complementarity in the 3′-UTR region and activation of the antisense strand (138). In contrast, CRISPR/Cas9 can achieve more stable suppression by permanently knocking out the target gene: in L-02 hepatocytes, Piezo1 deficiency alleviates stiffness-induced dysfunction and DNA damage, exerting a protective effect through the ERK1/2 pathway (139). In addition, researchers have constructed a C. elegans model carrying the disease-associated pezo-1[R2405P] mutation and, through whole-genome screening, found that gex-3 mutation can rescue reproductive defects, revealing the coupling relationship between Piezo channels and the F-actin network (140). Overall, gene-intervention strategies offer powerful tools for Piezo1-specific modulation and disease modeling, but off-target effects, the risks of permanent gene editing, and ethical concerns still limit their clinical application. In the future, lipid nanoparticles, ultrasmall micelles, or tumor-penetrating peptides may be employed as nano-delivery systems to achieve spatiotemporally controlled gene delivery and expression, thereby reducing potential risks while enhancing tissue specificity and promoting the clinical translation of Piezo1-targeted precision therapy.

### 5.4 Emerging strategies expanding the therapeutic scope of Piezo1 in cancer

In recent years, multidimensional innovative strategies have continuously broadened the application boundaries of Piezo1-targeted modulation in cancer therapy. These strategies not only include direct interventions on tumor cells but also encompass TME remodeling, drug delivery optimization, and enhancement of immune responses, reflecting a research trend of deep integration between mechanosensitive signaling and tumor biology. Firstly, in a breast cancer bone metastasis model, the traditional Chinese medicine formula Zuo Gui Wan (ZGP) modulates the secretion of TNF-α and IFN-γ by tumor cells, thereby inhibiting the overactivated Piezo1-GPX4 signaling in osteoblasts, blocking aberrant tumor-osteoblast communication, and upregulating PIK3CA expression to preserve osteoblast function. This reveals a novel molecular mechanism for traditional Chinese medicine intervention in bone metastasis (141). Secondly, the “cell backpack” technique directly attaches therapeutic payloads to the surface of DCs, activating Piezo1 channels via mechanical stimulation, which triggers cytoskeletal remodeling and type I interferon release, significantly enhancing antitumor immunity in combination with radiotherapy (142). In the drug delivery field, the arginine-rich undecapeptide R11 activates a Piezo1/integrin β1 positive feedback loop via membrane fusion, promoting Ca²^+^ influx and amplifying YAP signaling, which facilitates the formation of lamellipodia and enhances drug uptake efficiency, thereby improving targeted delivery to bladder cancer cells (64). Furthermore, elasticity-tunable silica nanoparticles (81–837 MPa) activate Piezo1 through membrane deformation, triggering NF-κB signaling and Ca²^+^ influx, leading to the repolarization of TAMs from M2 to M1 phenotype, and significantly suppressing tumor growth in hypoxic regions. This strategy introduces the concept of “nanomechanical immunoengineering” (143). The biomimetic metal Ti2448 stimulates Piezo1 on macrophages, suppresses LATS1/2-mediated YAP phosphorylation, and induces M1-to-M2 polarization while promoting the secretion of PDGF-BB and BMP-2, thereby enhancing angiogenesis and osteogenesis. This presents a new paradigm for the tri-modulation of implant materials, immunity, and tumors (100, 144). Therefore, emerging strategies involving Piezo1-focused traditional medicine, mechanical immunoengineering, targeted delivery systems, biomaterials, and nanomechanics collectively demonstrate the multidimensional value of mechanosensitive pathways in cancer therapy. These cutting-edge approaches provide a rich toolkit for personalized anticancer treatments and underscore the need for continued research into drug safety, tissue specificity, and mechanobiological coupling mechanisms to advance the clinical translation of Piezo1-targeted therapies.

## 6 Future perspectives

Although Piezo1 has demonstrated significant roles in cancer and the immune system, current research is still in its infancy and many issues remain unresolved. First, existing studies suggest that transient versus sustained activation of Piezo1 produces “biphasic” effects on immune polarization, yet the mechanical activation thresholds, Ca²^+^ peak–duration curves, and metabolic coupling profiles of different immune-cell subsets have not been systematically quantified. Key questions—such as whether Piezo1 regulation of the Treg/Th9 balance is time-dependent and whether distinct Piezo1 isoforms or splice variants possess cell-specific functions—have not been answered, limiting its application as a precise immunomodulatory target. Second, how the complex mechanical cues within the TME are integrated through Piezo1 to govern multiple downstream pathways remains to be fully elucidated. Third, although Piezo1 agonists and inhibitors such as Yoda1 and GsMTx4 have shown promise *in vitro*, their *in vivo* safety, specificity, and targeting require evaluation, and clinically applicable agents are lacking. Future work on Piezo1 in oncology and immunotherapy can expand in several directions: (1) using microfluidic, time-resolved single-cell imaging platforms to map Piezo1–Ca²^+^ activation dynamics against Foxp3/IL-9 expression phase diagrams, thereby defining whether an early pro-Th9 vs. late pro-Treg window exists with uniform temporal or cell-specific thresholds; (2) constructing multimodal three-dimensional mechanical-stimulation systems and applying phosphoproteomics to track real-time coupling of Piezo1–YAP/Calpain/NF-κB pathways, in order to determine which mechanical-parameter combinations dictate immune activation versus suppression; (3) developing Piezo1-targeted antitumor strategies by integrating gene-editing with nanomedicine technologies to inhibit the pro-tumor functions of tumor-specific high Piezo1 expression; (4) exploring combination therapy of Piezo1 modulation with immune-checkpoint inhibitors such as PD-1/PD-L1 or CTLA-4 to evaluate synergistic effects; and (5) dissecting interactions between the tumor mechanical landscape and Piezo1 using 3-D organoids and microfluidic chips to systematically assess how mechanical variations affect Piezo1 expression and function. Elucidating the integrated “threshold–time–splice” code of Piezo1 and deeply coupling it with tumor multiphysics and next-generation specific drugs will be pivotal for transforming Piezo1 from a “mechanistic star” into a bona fide clinical target, thereby enabling precision remodeling of cold TMEs.

## 7 Conclusion

Piezo1 is a central nexus connecting mechanosensation to cell-fate decisions throughout tumor initiation, progression, and immunoregulation. In tumor cells, Piezo1-mediated Ca²^+^ signaling promotes cytoskeletal remodeling, epithelial–mesenchymal transition, YAP/TAZ activation, and angiogenesis; concomitantly, Piezo1 drives cancer-associated fibroblast activation and ECM remodeling, collectively reinforcing invasion and therapy resistance. At the immune-cell level, Piezo1 modulates T-cell differentiation, dendritic-cell maturation, and macrophage polarization, potentially amplifying antitumor immunity yet, under chronic mechanical stress, also fostering immunosuppression. Thus, Piezo1 possesses dual “pro-tumor” and “immune-regulatory” attributes, forming a critical hub where mechanical signaling, tumor biology, and the immune milieu intersect. Comprehensive dissection of Piezo1 activation thresholds, temporal dependence, and splice-variant diversity—and development of highly specific modulators or precision delivery systems—will provide new strategies for integrating mechano-immunotherapy. Ultimately, Piezo1 is poised to become a powerful auxiliary target for converting cold TMEs, improving immunotherapy responsiveness, and overcoming therapeutic resistance.
